# Redox Profiling Reveals Clear Differences between Molecular Patterns of Wound Fluids from Acute and Chronic Wounds

**DOI:** 10.1155/2018/5286785

**Published:** 2018-11-18

**Authors:** Edina Bodnár, Edina Bakondi, Katalin Kovács, Csaba Hegedűs, Petra Lakatos, Agnieszka Robaszkiewicz, Zsolt Regdon, László Virág, Éva Szabó

**Affiliations:** ^1^Department of Dermatology, Faculty of Medicine, University of Debrecen, Debrecen, Hungary; ^2^Department of Medical Chemistry, Faculty of Medicine, University of Debrecen, Debrecen, Hungary; ^3^MTA-DE Cell Biology and Signaling Research Group, Debrecen, Hungary; ^4^Department of General Biophysics, University of Łódź, Łódź, Poland

## Abstract

Wound healing is a complex multiphase process which can be hampered by many factors including impaired local circulation, hypoxia, infection, malnutrition, immunosuppression, and metabolic dysregulation in diabetes. Redox dysregulation is a common feature of many skin diseases demonstrated by virtually all cell types in the skin with overproduction of reactive oxygen and nitrogen species. The objective of this study was to characterize the redox environment in wound fluids and sera from patients suffering from chronic leg ulcers (*n* = 19) and acute wounds (bulla fluids from second degree burns; *n* = 11) with serum data also compared to those from healthy volunteers (*n* = 7). Significantly higher concentrations of TNF-*α*, interleukine-8, vascular endothelial growth factor, and lactate dehydrogenase (measure of cell damage) were found in fluids from chronic wounds compared to acute ones. The extent of protein carbonylation (measure of protein oxidation), lipid peroxidation, and tyrosine nitration (indicator of peroxynitrite production) was similar in acute and chronic wound fluids, while radical scavenging activity and glutathione (GSH) levels were elevated in chronic wound fluids compared to acute wounds. Sera were also assessed for the same set of parameters with no significant differences detected. Nitrotyrosine (the footprint of the potent oxidant peroxynitrite) and poly(ADP-ribose) (the product of the DNA damage sensor enzyme PARP-1) could be detected in wound biopsies. Our data identify multiple signs of redox stress in chronic wounds with notable differences. In chronic wounds, elevations in antioxidant levels/activities may indicate compensatory mechanisms against inflammation. The presence of nitrotyrosine and poly(ADP-ribose) in tissues from venous leg ulcers indicate peroxynitrite production and PARP activation in chronic wounds.

## 1. Introduction

According to the World Health Organization (WHO), injuries account for 9% of global mortality with much higher numbers of associated hospitalizations and emergency department visits [1] compared to other causes of mortality. Many of the injuries are accompanied by skin wounds. While skin wounds caused by accidental or surgical cuts typically heal well, other types of dermatological ulcers (discontinuities of the skin) such as diabetic ulcers or pressure ulcers pose a real challenge in terms of successful wound management [2].

Wound healing can be delayed by various factors, including infections, impaired local circulation, hypoxia, venous stasis, nutritional problems, tension on wound edges, chronic inflammation, immunosuppression, and metabolic dysregulation due to diabetes. Because of the high incidence of diabetes in Western populations proper metabolic control of diabetics is of the utmost importance for the prevention of diabetic ulcers. Of note, 5% of diabetic patients develop foot ulcers and 1% require amputation [3].

Wound healing proceeds through four phases: hemostasis, inflammation, proliferation, and remodeling. Although the complex events of wound healing have been histologically well characterized, a comprehensive understanding of the biochemical and cellular events controlling normal and pathological wound healing is lacking.

Disruption of redox homeostasis is a common feature of various pathological conditions [4]. Reactive oxygen and nitrogen species (ROS and RNS, respectively) are produced by virtually all cells and tissues. Sources of ROS and RNS include the mitochondrial respiratory chain, autoxidation processes, NADPH oxidase, and nitric oxide synthase (NOS) enzymes. The physiological roles of ROS and RNS include but are not limited to host defense, exercise, vasorelaxation, oxygen sensing, and regulation of transcription and cell growth, only to name a few. The physiological signaling roles of ROS and RNS require redox sensor proteins such as activator protein 1 (AP-1, a heterodimer of c-Jun and c-Fos), heat shock factor 1 (HSF-1), or Keap-1. Redox switching relies on oxidation or glutathionylation of cysteine residues [5]. Nitric oxide, on the other hand, can regulate protein function by nitrosation or nitrosylation (e.g., of myocyte enhancer factor 2 (MEF-2) or guanylate cyclase, respectively) [6]. Moreover, an extensive antioxidant defense system consisting of small molecular and enzymatic antioxidants (e.g., glutathione, superoxide dismutase, catalase, and glutathione-peroxidase, respectively) operates to prevent tissue damage caused by ROS/RNS.

In contrast to redox regulation, oxidative stress due to an imbalance between production and elimination of ROS and RNS results in damage to proteins, lipids, and DNA. One particular oxidative stress pathway has received considerable attention. This oxidative stress pathway relies on the formation of nitric oxide-derived peroxynitrite (ONOO^−^), peroxynitrite-induced DNA strand breakage, and activation of the DNA break sensor enzyme poly(ADP-ribose) polymerase (PARP-1, ARTD1) [7]. Peroxynitrite is formed in a diffusion limited reaction between superoxide and nitric oxide:

(1) $$\begin{array}{c} {NO}^{\cdot }+{O_{2}}^{\cdot -}\to {ONOO}^{-} \end{array}$$

Besides triggering lipid peroxidation and protein oxidation and nitration, peroxynitrite is a potent inducer of DNA single strand breaks (SSB). PARP-1 is rapidly activated by DNA SSBs and cleaves NAD^+^ to nicotinamide and ADP-ribose. PARP1 then attaches ADP-ribose to suitable protein acceptors near the DNA nicks and builds a branched poly-ADP-ribose (PAR) polymer to initiate the repair process. PAR polymers and NAD consumption may induce cell dysfunction and cell death as demonstrated in various pathological conditions such as diabetic endothelial dysfunction [8], stroke, myocardial ischemia reperfusion injury, and many forms of inflammation. The nitric oxide-peroxynitrite-PARP activation pathway has also been implicated in the pathophysiology of many skin disorders. As for the involvement of this pathway in wound healing, we and others have demonstrated that PARP activation delays healing of various types of skin wounds by regulating keratinocyte migration, suppressing the production of inflammatory mediators, and promoting angiogenesis [9–12]. NO, on the other hand, has been shown to improve the histological, histochemical, and electron-microscopic characteristics of skin wounds in rats (Shekter AB et al. Nitric Oxide 2005). While NO may be beneficial, no such positive role for ONOO^−^ has been demonstrated in disease models: data obtained with peroxynitrite decomposition catalyst compounds point towards a damage-promoting role of this oxidant.

Human data on the role of redox imbalance, as well as the role of the nitric oxide-peroxynitrite-PARP-inflammation-wound healing axis, are scarce. Therefore, the aim of this study was to characterize the redox environment in acute and chronic wounds and to track elements of the peroxynitrite-PARP activation axis in human wounds. Our data show increased inflammatory activity and upregulated antioxidant homeostasis in chronic wounds compared to acute ones. Moreover, we found that peroxynitrite and PARylation are active in chronic wounds.

## 2. Methods

### 2.1. Human Samples

The protocol for collecting human samples was in accordance with the Declaration of Helsinki and was approved by the Regional Research Ethics Committee (RKEB 2695). Patients signed informed consents. Patient data (gender, age, wound type, and wound area) are presented in Table 1. Wound fluids were collected from a total of 30 patients. Wounds of patients with chronic ulcers (venous leg ulcers (*n* = 16) and diabetic ulcers (*n* = 3)) were covered with film bandages, and wound fluids accumulating over 12 h were collected from underneath the bandage with a sterile needle and syringe. Fluids from acute wounds were collected from bullas of patients (*n* = 11) with second degree burns by aspirating the fluid with a sterile needle. Samples from both chronic and acute wounds were centrifuged (2000xg, 30 min), and supernatants were stored at −70°C. Serum samples were obtained from the peripheral blood of all 30 patients as well as from healthy controls (*n* = 7) by routine technique. Healthy controls underwent minor surgery to excise benign skin tumors. Tissue samples were obtained from the chronic leg ulcers (*n* = 14) in order to exclude spinocellular carcinoma. Two of the total 16 leg ulcer patients did not consent to biopsy. All 14 samples were devoid of tumor. Normal skin samples were also obtained from the healthy controls.

### 2.2. Detection of Nitrotyrosine in Tissues

Nitrotyrosine was detected in formaldehyde-fixed and paraffin-embedded sections as previously described [13]. The rabbit polyclonal anti-nitrotyrosine antibody (Merck, Budapest, Hungary) was applied at 1 : 1000 dilution. The secondary antibody was a biotinylated goat anti-rabbit IgG (supplied with the Vector Elite kits; Vector Laboratories, Burlingame, CA). The ABC reagent was prepared and used as recommended by the manufacturer. Color was developed with nickel-enhanced DAB substrate followed by counterstaining with chromotrope dye.

### 2.3. Detection of PARP Activity and PAR Polymer in Tissues

Poly(ADP-ribose) the polymer product of the PARP-catalyzed reaction was detected with standard immunohistochemical procedure as previously described [14]. Nitrotyrosine and PAR stainings were semiquantitatively evaluated by assigning to each section a visual score (from 0 to three) as follows: 0: negative, 1: less than ¼ of cells showed weak positivity, 2: 25–50% of cells displayed varying degrees of positivity, and 3: majority of cells showed moderate to strong positivity.

### 2.4. Assessing Radical Scavenging Activity of Wound Fluids

The antioxidant capacity of wound fluids was assessed with three methods as detailed below.

#### 2.4.1. Measurement of the Radical Scavenging with ABTS Decolorization Assay

Measurement of the radical scavenging activity in samples was performed with the ABTS (2,2′-azinobis-(3-ethylbenzothiazoline-6-sulfonic acid)) decolorization assay as described previously [15]. ABTS^+^ radical cation was generated by oxidation of ABTS with potassium persulfate at room temperature overnight in the dark. The next day, shortly before the experiment, absorbance of the ABTS^+^ solution was adjusted with Gly-HCl buffer to 1.2 at 405 nm. Samples were diluted 2-fold with PBS and were then incubated with ABTS^+^ solution for 30 minutes in 96-well plates in triplicate. Absorbance was measured with Victor V [3] multilabel reader (405 nm). Decolorization (i.e., antioxidant capacity) was expressed as a percentage of control. Positive controls were 3 mM NAC and 10 *μ*M ascorbic acid, both displaying 100% antioxidant capacity.

#### 2.4.2. Cupric Ion Reducing Antioxidant Capacity (CUPRAC) Assay

The CUPRAC assay utilizes copper(II)-neocuproine (Cu(II)-Nc) reagent as the chromogenic oxidant, which changes the maximum absorbance after reduction with antioxidants. Measurement of the antioxidant capacity of wound fluids was performed as described by Apak et al. [16] with the following modifications: 12 *μ*M of Trolox was used as a positive control, the final volume was reduced to 100 *μ*L, and the measurements were performed in 96-well microplates.

#### 2.4.3. Determination of H_2_O_2_ Scavenging Activity in Amplex Red Assay

Hydrogen-peroxide scavenging capacity was measured using the Amplex Red reagent (Thermo Fisher Scientific). Serum or wound fluid/drainage samples were incubated in 1000x dilution with 1 *μ*M H_2_O_2_ (Sigma-Aldrich), 50 *μ*M Amplex Red reagent, and 0.1 U/mL horseradish peroxidase (Sigma-Aldrich) in phosphate buffered saline for 30 minutes at room temperature. For each serum sample, serum blanks were prepared. In the presence of horseradish peroxidase, H_2_O_2_ reacts stoichiometrically with the Amplex Red reagent (10-acetyl-3,7-dihydroxyphenoxazine) to generate the red-fluorescent oxidation product, resorufin. Fluorescence was read with excitation at 530 nm and emission at 590 nm using a Fluoroskan Ascent FL plate reader (Labsystems, Vantaa, Finland).

### 2.5. Glutathione (GSH)

Serum was precipitated for 15 minutes with RQB-TCA (10%) solution on ice. After centrifugation (5000g, 15 min, 4°C), the supernatant was collected for further analysis. For glutathione estimation, 5 *μ*L of the supernatant was put into a 96-well plate, followed by addition of 40 *μ*L of 1 M potassium phosphate buffer. After shaking and a 5 minute incubation at room temperature, 140 *μ*L of potassium phosphate buffer was added, followed by 25 *μ*L of 0.5% o-phthalaldehyde. The sample was shaken and incubated for 30 minutes at room temperature. Fluorescence was read at excitation 390 nm and emission 460 nm. As a background for each sample, appropriate controls were used: supernatant and buffer incubated with 4 *μ*L of 7.5 mM N-ethylmaleimide for 30 minutes at room temperature. Standard curves were prepared with glutathione. Protein concentration was determined using the bicinchoninic acid (BCA) method.

### 2.6. Determination of Cytokines in Wound Fluids

Amounts of inflammatory proteins were determined in sandwich ELISA formats using the corresponding Human Mini TMB ELISA Development Kit (PeproTech, USA) following the manufacturer's instructions.

### 2.7. Quantitation of Protein Carbonylation as a Marker of Protein Oxidation

From each sample, 20 *μ*L was diluted with distilled water to 200 *μ*L. After addition of 50 *μ*L of 80% TCA solution, each sample was vortexed and incubated on ice for 5 minutes. After centrifugation at 13,000xg for 2 minutes, supernatants were removed and pellets were resuspended in 500 *μ*L ice-cold acetone. Incubation for 5 minutes at −20°C was followed by another centrifugation at 13,000xg for 2 minutes. Acetone was removed, pellets were dissolved in 20 *μ*L distilled water, and 15 *μ*L of sample was used for further protein carbonyl analysis using OxyBlot Protein Oxidation Kit (Millipore) following the manufacturer's instructions. After completion of the derivatization step, the obtained DNP product yield was quantified spectrophotometrically by measuring absorbance at 405 nm.

### 2.8. Quantitation of the Lipid Peroxidation Products (Thiobarbituric Acid Reactive Species, TBARS)

Samples (10 *μ*L) were transferred into microcentrifuge tubes followed by addition of 10 *μ*L 8.1% SDS, 75 *μ*L 20% acetic acid, 75 *μ*L TBA (thiobarbituric acid), and 30 *μ*L dH_2_O. The reaction mixtures were incubated at 95°C for 45 min, then 100 *μ*L of each reaction mixture was pipetted into individual wells of a 96-well plate. Absorbance was read at 532 nm.

### 2.9. Protein Tyrosine Nitration

Protein content of samples were estimated by BCA reagent. Protein concentration in each sample was diluted to 20 *μ*g/*μ*L. Before the experiment, nitrocellulose membrane was activated by TBST (50 mM Tris, 0.5 M NaCl, 0.05% Tween 20, pH 7.4) for 20 minute at room temperature. Samples (1 *μ*L) were spotted onto the membrane in 3 different dilutions (100x, 500x, and 1000x). The membrane was blocked in 5% nonfat dry milk in TBST for 1 hour at room temperature. The membrane was then incubated with an anti-nitrotyrosine primary antibody (Merck Millipore), which was diluted in 1% milk in TBST overnight at 4°C. After washing three times with TBST, the membrane was incubated with peroxidase-conjugated secondary antibody (goat anti-rabbit IgG, Cell Signaling Technology) diluted in 1% milk in TBST for 1 hour at room temperature. To visualize the antibody reaction, chemiluminescent substrates were added, and chemiluminescent signals were detected using a FluorChem FC2 Imager. Densitometric analysis was carried out using ImageJ software. Nitrotyrosine values were expressed in mol/*μ*g protein calculated from the values of peroxynitrite-treated BSA, which were used as a positive control.

### 2.10. Lactate Dehydrogenase Activity

Lactate dehydrogenase released from damaged cells was measured with a commercial kit (Roche Applied Science, Mannheim, Germany). Samples (100 *μ*L) were transferred to 96-well flat bottom plates. Detection reagent (100 *μ*L) was added, plates were incubated (30 min), and the absorbance of samples was measured at 490 nm.

### 2.11. Statistical Analysis

Primary data are presented on box and whisker plots showing the medians, the lower and higher quartiles, and minimum and maximum values. Statistical significance was determined using *t* tests. Significance of the relationship between variables and corresponding correlation coefficients were determined using Spearman two-tailed correlation analysis tests performed with SigmaPlot software. *P* values of less than 0.05 were considered significant.

## 3. Results

In order to assess redox homeostasis in wound patients, we have determined footprints of ROS/RNS species and characterized antioxidant homeostasis in wound fluids and in sera. ROS and RNS cause many different types of alterations in biomolecules. Most ROS/RNS species can oxidize proteins resulting in protein carbonylation whereas oxidative lipid modifications result in lipid peroxidation. Antioxidant homeostasis was assessed by determining radical scavenging activities and levels of the main antioxidant molecule glutathione.

**Figure 1:**
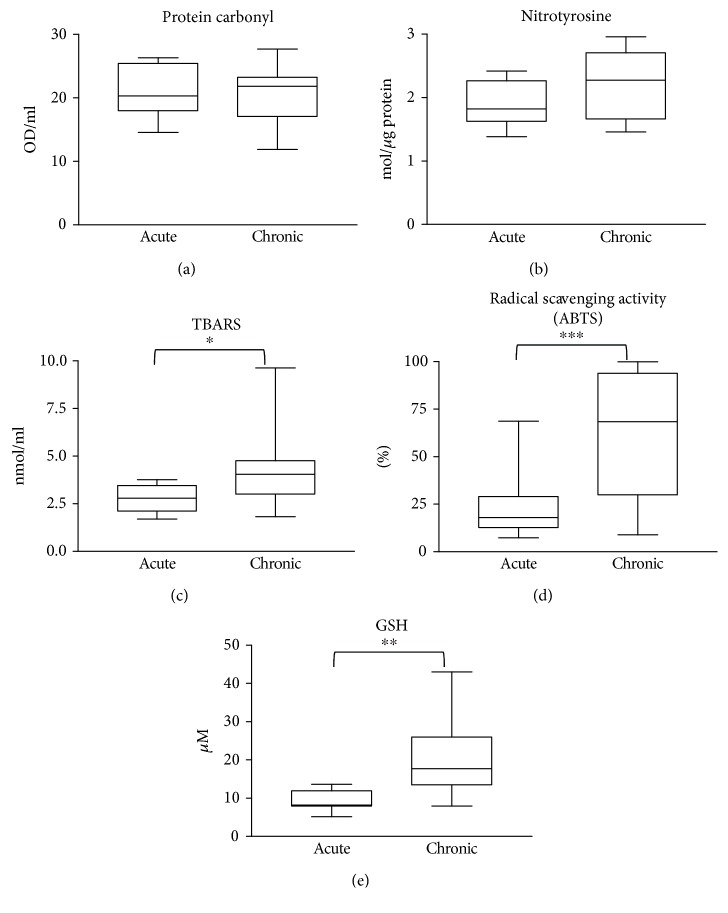
Biomolecular damage and antioxidant status in the wound fluids. Wound fluids from acute (burn) wounds and from chronic wounds of venous leg ulcer and diabetic ulcer patients were compared with respect to protein oxidation (a), protein tyrosine nitration (b), and lipid peroxidation (c). Total antioxidant activity was assessed with ABTS decolorization assay (d), and the levels of the main thiol antioxidant glutathione were also determined (e). (^∗^ *p* < 0.05; ^∗∗^ *p* < 0.01; ^∗∗∗^ *p* < 0.001).

**Figure 2:**
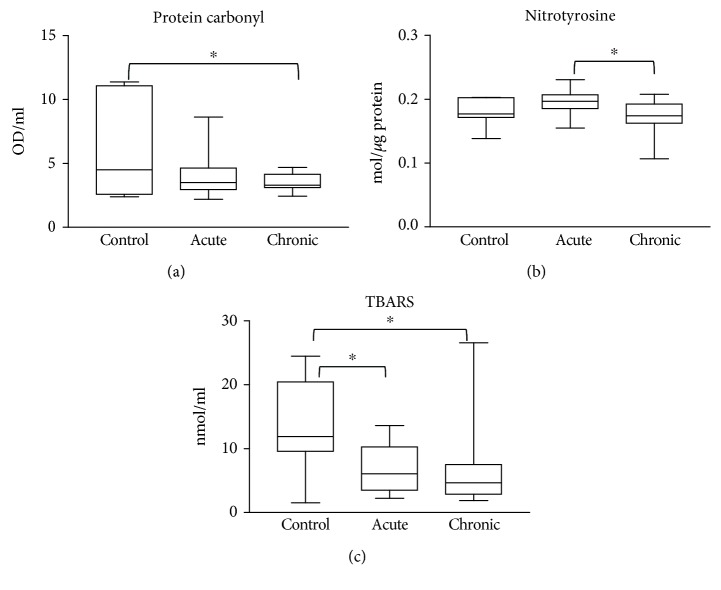
Biomolecular damage markers in the sera of patients. Sera from healthy controls, burn patients (acute), venous leg ulcer and diabetic ulcer patients (chronic) were compared with respect to protein oxidation (a), protein tyrosine nitration (b), and lipid peroxidation (c). No significant differences were detected between groups.

**Figure 3:**
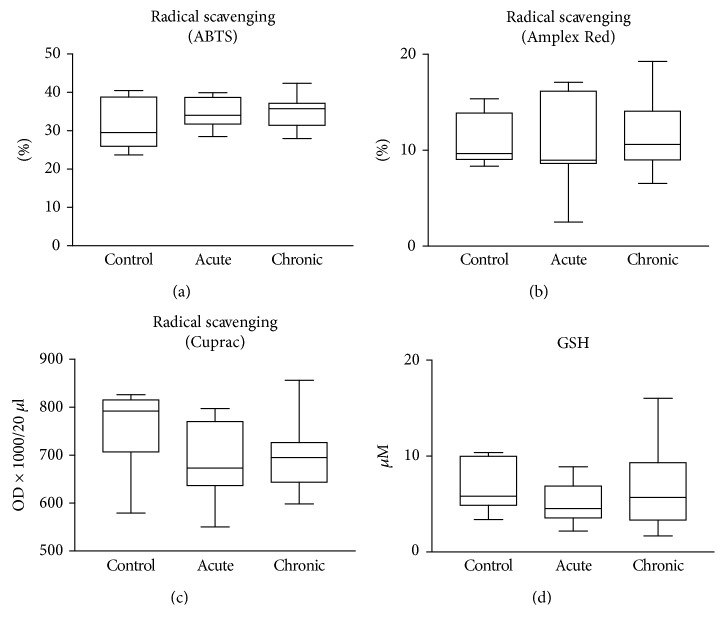
Antioxidant status in the sera of patients. Sera from healthy individuals (control), burn patients (acute), venous leg ulcer and diabetic ulcer patients (chronic) were compared with respect to antioxidant status. Antioxidant activity was assessed with ABTS decolorization assay (a), Amplex Red assay (b), and Cuprac assay (c). The levels of the main thiol antioxidant, glutathione, was also determined (d). No significant differences were detected between the groups.

We found detectable levels of biomolecular damage in wound fluids of venous leg ulcers (Figure 1). However, there were no significant differences in protein oxidation and protein tyrosine nitration between the two patient groups (acute and chronic wounds) (Figures 1(a) and 1(b)). Lipid peroxidation was slightly but significantly higher in chronic wound samples compared to acute ones (Figure 1(c)). Moreover, radical scavenging activity as well as levels of the main antioxidant glutathione were significantly different; chronic wounds contained considerably more antioxidants compared to acute wound fluids (Figures 1(d) and 1(e)). In sera from wound patients, no major differences could be seen between sera from acute and chronic ulcer patients. However, levels of protein carbonylation and lipid peroxidation products were slightly but significantly lower in sera of chronic wound patients compared to healthy controls (Figures 2(a) and 2(c)). Lipid peroxidation products were also lower in sera from acute wound patients compared to control. Moreover, no major difference could be detected in protein tyrosine nitration in sera from controls and wound patients, but sera from chronic wound patients showed slightly lower nitrotyrosine levels compared to acute ones (Figure 2(b)). Antioxidant levels in the sera of acute or chronic wound patients were not different from those of healthy controls (Figure 3).

**Figure 4:**
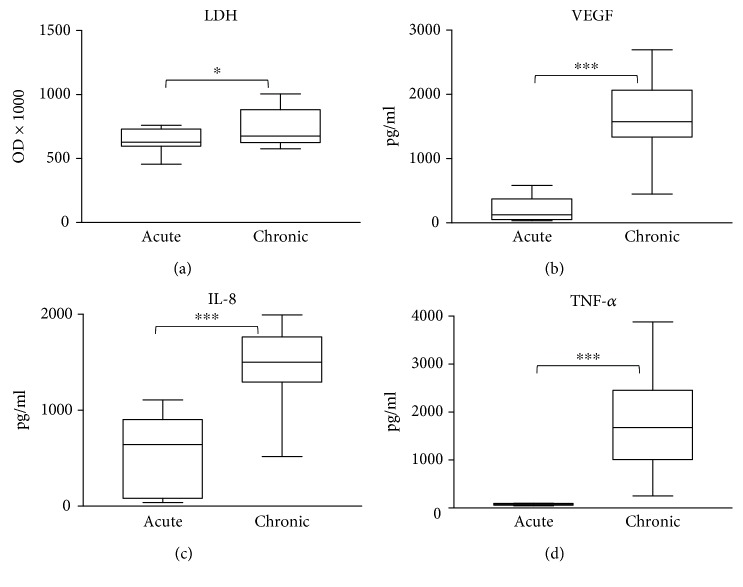
Markers of chemoattraction, inflammation, vascularization, and tissue damage in wound fluids. Wound fluids from venous ulcers and diabetic wounds (chronic) and burn wounds (acute) were analyzed for markers of (a) tissue damage (LDH), (b) vascularization (VEGF), (c) granulocyte chemoattraction (IL-8), and (d) inflammation (TNF-*α*). (^∗∗^ *p* < 0.01; ^∗∗∗^ *p* < 0.001).

Lactate dehydrogenase (LDH) activity indicating tissue injury, mediators of granulocyte recruitment (IL-8), inflammation (TNF-*α*), and vascularization (VEGF) were significantly higher in the wound fluids of chronic wound patients compared to acute ones (Figure 4(a)–(d)). These biomarkers were not detectable in the sera of patients (not shown).

**Figure 5:**
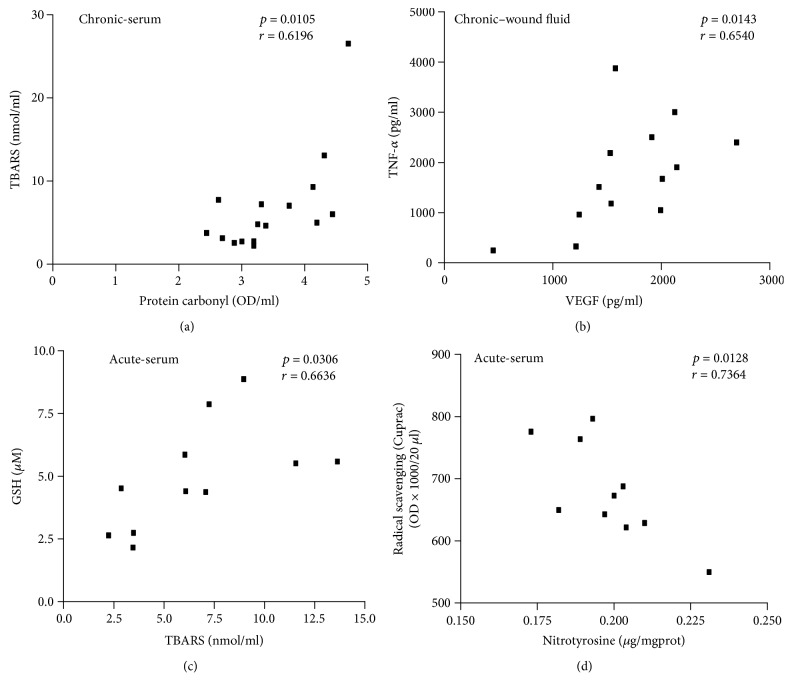
Correlation analysis of wound fluid and serum biomarkers. Pairwise correlations were performed on all parameters in the study. Four pairs of parameters yielding significant positive ((a)–(c)) or negative (d) correlations are shown.

A pairwise correlation of all measured biochemical parameters was also performed. A significant correlation was established in the sera of chronic wound patients between protein carbonylation and lipid peroxidation (Figure 5(a)). Moreover, in chronic wound fluids, VEGF and TNF-*α* levels also correlated significantly with each other (Figure 5(b)). Furthermore, in the sera of acute wound patients, a positive correlation was found between lipid peroxidation and glutathione levels (Figure 5(c)), while an inverse correlation could be established between tyrosine nitration and radical scavenging activity (Figure 5(d)).

### 3.1. Detection of Nitrotyrosine and Poly(ADP-Ribose) (PAR) in Tissue Samples

**Figure 6:**
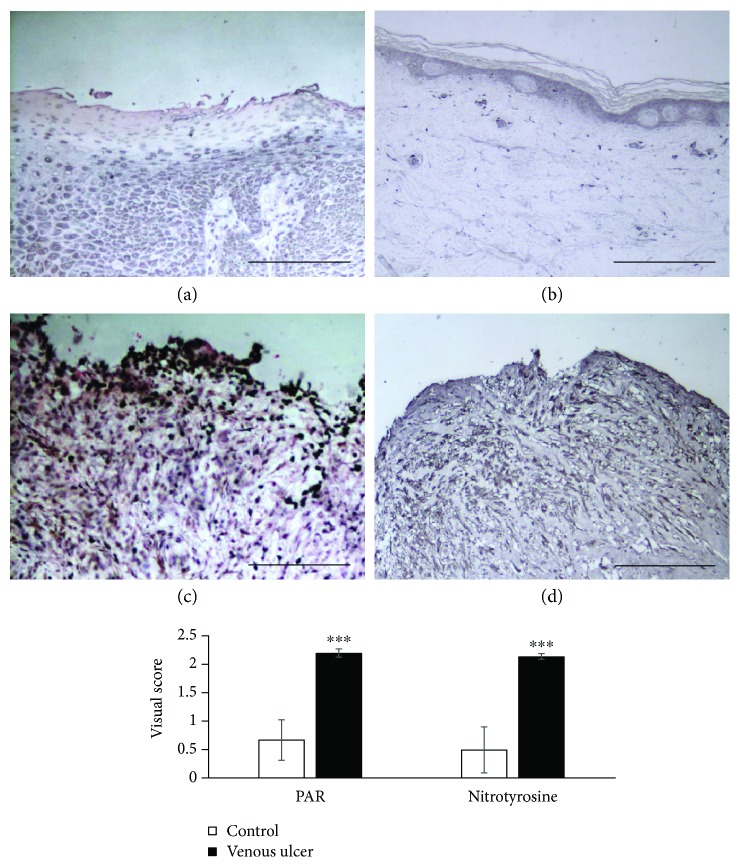
Immunohistochemical detection of nitrotyrosine and poly(ADP-ribose) in ulcer biopsies. Tissue biopsies from healthy skin ((a), (b)) and chronic venous ulcers ((c), (d)) were stained for poly(ADP-ribose) ((a), (c)) and nitrotyrosine ((b), (d)). Scale bars represent 200 *μ*m. Immunostainings were evaluated by a semiquantitative visual scoring, as described in the Methods section. (^∗∗∗^ *p* < 0.001).

Tissue biopsies were obtained from the wound area of venous leg ulcer patients in order to exclude the occurrence of malignancy (malignancies were not detected in any of the tissue samples). Sections from these tissues were stained for nitrotyrosine and PAR in order to verify *in vivo* production of peroxynitrite (the footprint of which is nitrotyrosine) and activation of PARP-1 (the product of which is PAR polymer). We found that wound areas in samples from venous leg ulcer patients extensively stained positive for both nitrotyrosine and PAR (Figures 6(c) and 6(d)). Healthy skin areas of patients who had no ulcer and underwent biopsies for naevus removal had no staining or weak positive staining for nitrotyrosine and PAR (Figure 6(a), 6(b)). Semiquantitative analysis revealed significantly elevated nitrotyrosine and PAR staining in the wound beds and surrounding tissue compared to tissues from healthy controls (Figure 6(e)).

## 4. Discussion

The wound healing process requires a highly orchestrated sequence of events carefully coordinated both in time and space. Reactive oxygen and nitrogen intermediates have been implicated in the regulation of wound healing, both promoting and inhibiting healing, according to published studies [17]. For example, ROS/RNS species play a crucial signaling role in the healing process (e.g., in cell proliferation and angiogenesis) and also contribute to defense against invading pathogens. In contrast, oxidative stress resulting from an imbalance between the production and elimination of reactive species clearly contributes to delayed healing of wounds, as observed in patients with diabetes or patients treated with chemotherapy, radiotherapy, or anti-inflammatory glucocorticoids. How and why the redox environment in acute and chronic wounds differ from each other is not fully understood. Furthermore, the mechanism by which changes in the redox environment in wounded tissues are mirrored in the serum is unknown.

ROS/RNS species may hit important biological targets (proteins, lipids, and nucleic acids) in tissues and leave their footprints on biomolecules. Many ROS/RNS species are oxidants and can cause protein oxidation in tissues. We have detected protein carbonylation (a protein oxidation marker) in the wound fluids from acute and chronic wounds with no significant difference between the acute and chronic conditions. Lipid peroxidation was slightly more intense in chronic wound fluids while tyrosine nitration (for example, caused by peroxynitrite-induced protein modification) was not different between the two groups. The antioxidant profile of acute and chronic wounds; however, differed significantly: chronic wounds had higher radical scavenging activity and higher GSH content compared to acute wounds. In parallel, levels of VEGF, a key mediator of vascularization, IL-8, the main granulocyte recruitment factor, and TNF-*α*, an inflammatory cytokine, had higher levels in chronic compared to acute wounds. These data collectively suggest a more profound inflammatory environment in the chronic wounds which is likely to be accompanied by higher production of ROS/RNS species. A compensatory overproduction of antioxidants (Figures 1(a) and 1(e)) may explain why increased ROS/RNS production is not reflected in accelerated biomolecule damage (Figures 1(a)–1(c)). The slightly but significantly higher lipid peroxide levels in chronic wound fluids likely indicate that the lipids may represent more vulnerable targets than proteins.

Molecular events taking place in diseased tissues are often detectable in the bloodstream, setting the stage for the widespread use of laboratory blood tests. We wondered if changes in tissue redox environment are mirrored by similar changes in the sera of patients. The lack of any detectable differences in radical scavenging activity, glutathione level, protein oxidation, and lipid peroxidation in the sera of patients hospitalized for chronic vs. acute wounds (Figures 2 and 3) suggests that differences in redox perturbations remain localized in and around the wound area without causing major differences between acute and chronic wound patients at the systemic level. Of note, lipid peroxidation products displayed lower levels in both acute and chronic patients' sera compared to controls, which is in sharp contrast to differences in opposite signs seen in wound fluids. It is likely that wound fluids directly mirror tissue level biochemical processes, while serum levels are more affected by clearance mechanisms.

Interestingly, pairwise correlation analysis of the measured biochemical parameters revealed significant correlations in some pairs (Figure 5). Of note, a positive correlation between two biomolecular footprints, namely, lipid peroxides and protein carbonyls, may suggest that the same kind of oxidative redox environment may trigger these two modifications in the sera of venous ulcer patients (Figure 5(a)). An inverse correlation between radical scavenging activity and nitrotyrosine formation indicates that radical scavengers may inhibit tyrosine nitration in the sera of acute wound patients (Figure 5(d)). The positive correlation between lipid peroxidation and glutathione in the sera of acute wound patients (Figure 5(c)) is somewhat surprising. In numerous oxidative stress-related conditions, lipid peroxidation typically increases while glutathione levels drop in the inflamed tissues [18]. Other studies reported a lack of parallel changes in GSH and lipid peroxide levels [19] indicating a much more complex relationship between these parameters. In contrast, the reducing effects of the glutathione “fragments” cysteinylglycine and cysteine have been shown to contribute to lipid peroxidation induced by a ferric ion chelate [20]. If levels of these thiols display changes similar to glutathione, this may explain why GSH and lipid peroxide levels change in parallel with each other in the sera of acute wound patients. However, the possibility that the correlation does not indicate causal relationship between the two parameters cannot be excluded.

Although VEGF expression is primarily regulated by hypoxia signaling [21], inflammatory cytokines such as TNF-*α* and IL-1*β* and oxidants such as H_2_O_2_ have also been reported to cause upregulation of VEGF [22, 23]. H_2_O_2_-induced VEGF expression has been shown to occur in the skin where it promotes wound healing [23]. VEGF induction by H_2_O_2_ appears to be independent of the hypoxia pathway but inhibitable by the thiol antioxidant N-acetylcysteine [22, 23]. In our chronic wound fluid samples, TNF-*α* levels correlated with VEGF levels. Thus, it seems plausible to hypothesize that inflammatory and oxidative signaling induce VEGF expression in chronic wounds. Nonetheless, deciphering the relationship between redox signaling and VEGF expression requires further investigation.

While analyzing differences in the redox environment of acute versus chronic wounds, one should not ignore the fact that our study populations had different age distributions. Our patient population with chronic wounds had an average age of 66.5, while the acute wound population had an average age of 45.4 years. Since the redox environment may change with age [24, 25], we could exclude the possibility that some differences between the two study groups were due to the difference in the average age of patients. In fact, some of the redox and inflammatory parameters measured in the study (ABTS scavenger activity of the serum and TNF-*α*, IL8, and VEGF levels in the wound fluid) correlated with age in the total study population (Supplementary Figure S2). From these parameters, serum ABTS activity showed no difference between the study groups (Figure 3(a)). Since other oxidative damage parameters showed no age dependency, we consider it unlikely that the observed signs of redox stress in the wound fluids were due to the age factor. Levels of TNF-*α*, IL8, and VEGF, on the other hand, correlated positively with age and showed higher values in chronic compared to acute wound samples. Further investigation with aged-matched study groups to confirm or disprove the role of age in the higher TNF-*α*, IL8, and VEGF levels of the chronic wound fluids compared to acute ones may be required.

Peroxynitrite formation occurs in many forms of inflammation [7]. Our data also prove that peroxynitrite is formed in human wounds as indicated by detectable levels of nitrotyrosine. Immunohistochemistry revealed increased tyrosine nitration in chronic wounds compared to healthy skin (Figure 6). This is likely due to the upregulated expression of inducible nitric oxide synthase [26] in parallel to elevated superoxide production [27]. Peroxynitrite and hydroxyl radicals efficiently break DNA strands leading to the activation of PARP-1 [7]. PARP activation in human chronic wounds was demonstrated by immunodetection of the enzyme's product, poly(ADP-ribose). Increased PAR polymer is thought to be mainly due to the activation of basally expressed PARP-1. However, PARP-1 expression is also upregulated in the wounds, especially in the wound edges (Supplementary Figure S1), and contributes to tissue' PARylation capacity. Increased PARylation may signal DNA repair following oxidative DNA damage, but may also contribute to cell damage in severe oxidative stress. The exact role of PARylation in human wounds requires further investigation.

Overall, we provided a detailed redox characterization of human chronic and acute wounds. To interpret the differences between these two types of wound fluids, one needs to be aware of the following caveats. While the chronic wounds from which we collected wound fluids were exposed to the outer environment (apart from the covering bandage) and must have been populated with their own microbiome, the blister fluids that served to represent acute wounds were covered with the blister roof and were sterile. Since our burn patients typically reached the burn unit within one hour and their blister fluids were removed within four hours, composition of these wound fluids were likely to differ from those obtained at later time points. Moreover, this early drainage of blisters also prevented reliable differentiation between superficial and partial thickness burns. According to the literature, wound fluid compositions from these two categories may not be identical [28]. Nonetheless, the redox parameters measured from these acute wounds displayed relatively little variations suggesting that our selected burn patient population was likely to be quite homogeneous. Other potential sources of wound fluid (surgical wounds or donor sites of skin grafts) usually do not provide sufficient sample volume for the high number of assays used in our study. We also rejected the idea of using fluids from open wounds, as in our dermatosurgery practice, only wounds healing by secondary intention are treated openly and these are all infected wounds.

In conclusion, our data prove that the redox environment in chronic human wounds clearly differs from acute wounds as reflected by intense inflammation accompanied by higher antioxidant levels. Detailed follow-up studies dissecting the roles of the individual ROS species and redox signaling mechanisms operating in chronic wounds may permit more precisely targeted pharmacological interventions for the treatment of delayed wound healing in the future. Moreover, whether a detailed redox and inflammatory biomarker profiling of wound fluids or sera can provide predictive biomarkers for the identification of nonhealing wounds remains to be seen. Peroxynitrite production and PARylation also occur in chronic wounds, and likely mediate tissue injury. The precise role of these processes in normal or pathological wound healing may be the subject of further investigations.

## Figures and Tables

**Table 1:** Clinical data of patients involved in this study.

Patient ID	Gender	Age	Wound type	Wound persisted for	Total wound area (cm^2^)
Patient data					
1.	f	85	Chronic venous	3 years	96
2.	f	70	Chronic venous	6 years	378
3.	m	54	Chronic venous	5 years	656
4.	f	60	Chronic venous	4 years	56
5.	m	82	Chronic venous	3 years	327.5
6.	m	69	Chronic venous	40 years	750
7.	f	70	Chronic venous	4 years	447
8.	m	83	Chronic venous	20 years	318
9.	m	66	Chronic venous	3 years	18
10.	m	52	Chronic venous	9 months	104
11.	f	82	Chronic venous	2 years	116
12.	f	40	Chronic venous	4 years	56
13.	f	70	Chronic venous	20 years	32
14.	f	68	Chronic venous	4 years	60
15.	m	65	Chronic venous	1.5 years	180
16.	m	46	Chronic venous	3 years	300
17.	m	52	Chronic diabetic	6 months	80
18.	m	68	Chronic diabetic	1 month	36
19.	f	81	Chronic diabetic	4 years	148
20.	f	44	Acute (2nd degree burn)	<4hours	1500
21.	f	61	Acute (2nd degree burn)	<4hours	225
22.	m	42	Acute (2nd degree burn)	<4hours	1800
23.	m	68	Acute (2nd degree burn)	<4hours	3000
24.	m	17	Acute (2nd degree burn)	<4hours	1950
25.	m	47	Acute (2nd degree burn)	<4hours	150
26.	f	71	Acute (2nd degree burn)	<4hours	300
27.	f	77	Acute (2nd degree burn)	<4hours	2250
28.	m	31	Acute (2nd degree burn)	<4hours	1500
29.	f	18	Acute (2nd degree burn)	<4hours	1500
30.	m	23	Acute (2nd degree burn)	<4hours	3750

Thirty patients were enrolled in the study; 19 with chronic leg ulcers and 11 patients with second degree burns. In the chronic wound group, 16 patients had venous ulcers and 3 had diabetic ulcers. Clinical data (age, sex, wound type, persistence, and total area of wounds) are presented.

## Data Availability

The data used to support the findings of this study are available from the corresponding author upon request.

## Supplementary Materials

Supplementary Materials Supplementary Figure S1: immunohistochemical detection of poly(ADP-ribose) polymerase-1 in ulcer biopsies. A tissue biopsy from a chronic venous ulcer was stained for poly(ADP-ribose) polymerase-1 (PARP-1). While wound edges displayed intense immunopositivity for PARP-1, nearby healthy skin showed weaker PARP-1 expression. The scale bar represents 200 *μ*m. Supplementary Figure S2: correlation analysis of wound fluid/serum biomarkers and patients' age. Correlation was analyzed between the age of patients and all biochemical parameters measured in the study. Four pairs of parameters yielding significant positive correlations are shown.
