# Supplementary figures and images for: Redox Profiling Reveals Clear Differences between Molecular Patterns of Wound Fluids from Acute and Chronic Wounds
Source: Oxid Med Cell Longev. 2018 Nov 18;2018:5286785. doi: 10.1155/2018/5286785 (PMC6276414; doi:10.1155/2018/5286785)

**Figure S1**

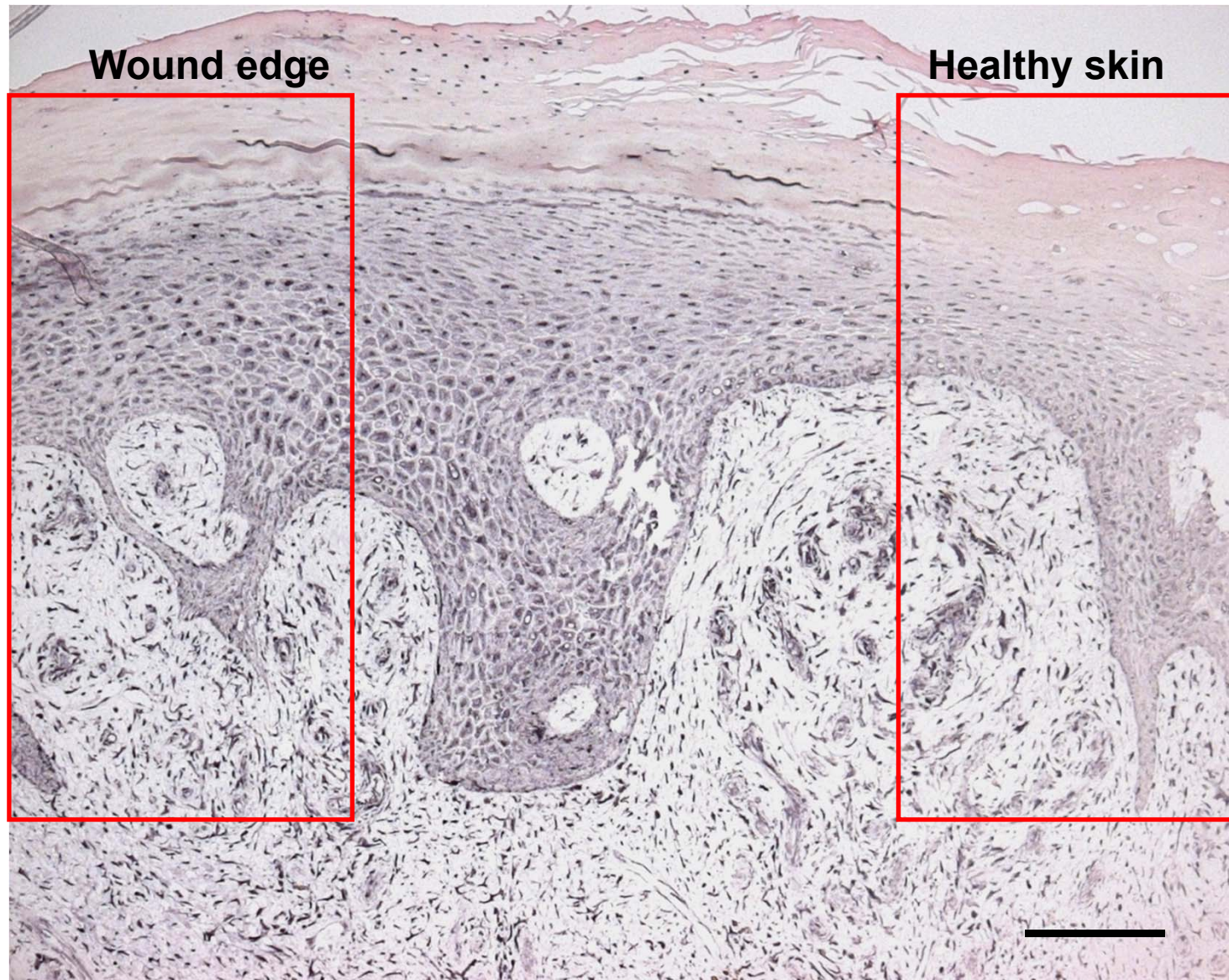

**Figure S2**

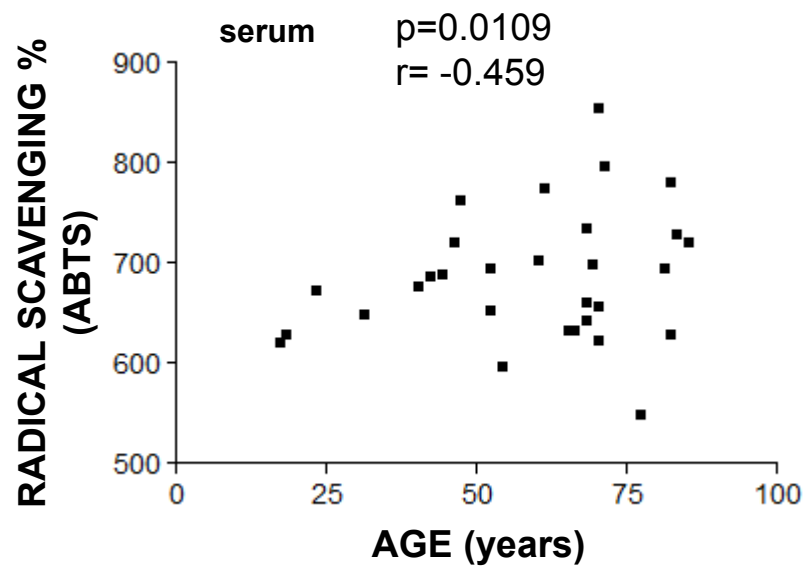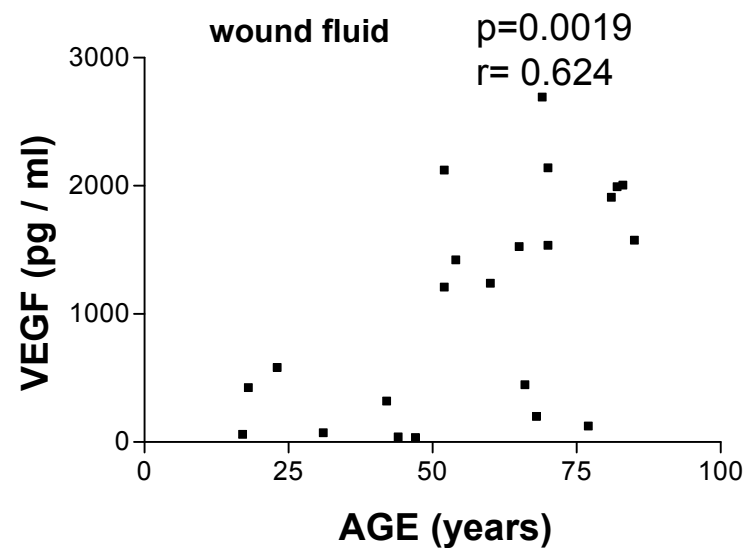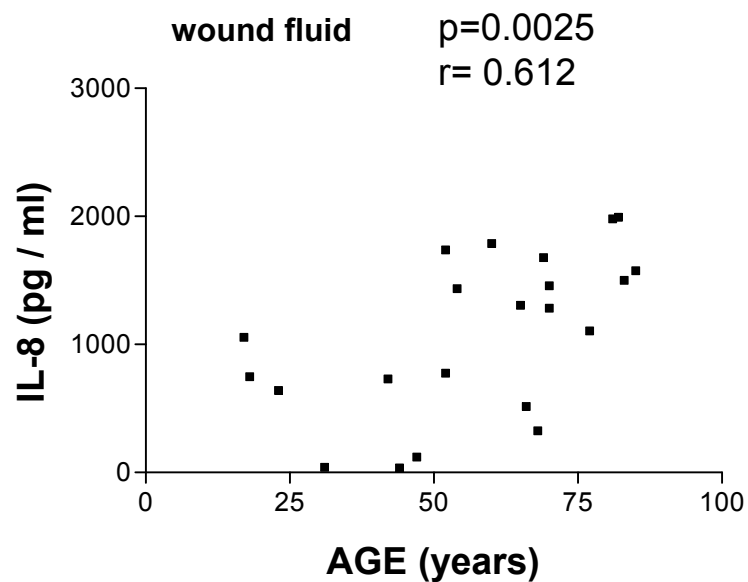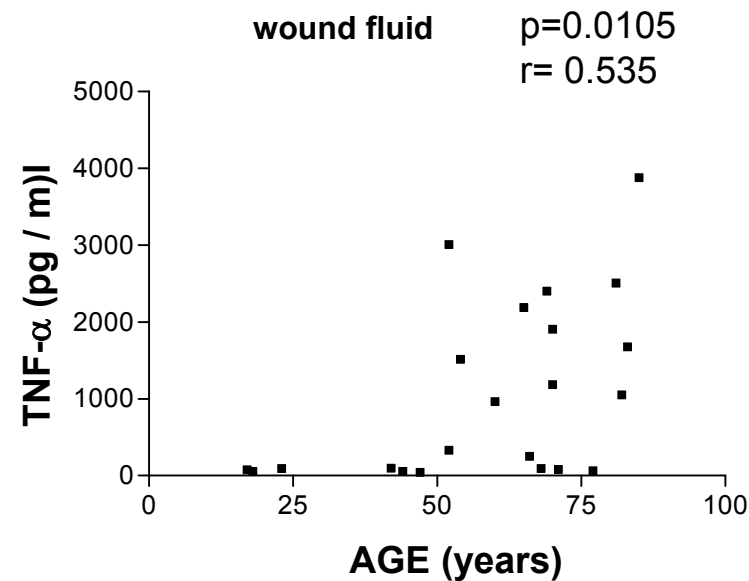

Supplement: Supplementary Materials — Supplementary Figure S1: immunohistochemical detection of poly(ADP-ribose) polymerase-1 in ulcer biopsies. A tissue biopsy from a chronic venous ulcer was stained for poly(ADP-ribose) polymerase-1 (PARP-1). While wound edges displayed intense immunopositivity for PARP-1, nearby healthy skin showed weaker PARP-1 expression. The scale bar represents 200 μm. Supplementary Figure S2: correlation analysis of wound fluid/serum biomarkers and patients' age. Correlation was analyzed between the age of patients and all biochemical parameters measured in the study. Four pairs of parameters yielding significant positive correlations are shown. [file 5286785.f1.pdf]
